# Supplementary material for: Purpuramine R, a New Bromotyrosine Isolated from Pseudoceratina cf. verrucosa Collected in the Kingdom of Tonga
Source: Mar Drugs. 2025 Apr 27;23(5):186. doi: 10.3390/md23050186 (PMC12113617; doi:10.3390/md23050186)
Supplement: Supplementary file 1 [file marinedrugs-23-00186-s001.zip › marinedrugs-3585474-supplementary.pdf]

Article

Purpuramine R, a new bromotyrosine isolated from *Pseudoceratina* cf. verrucosa collected in the Kingdom of Tonga

Jennie L. Ramirez-Garcia,[1,2] Hannah Lee-Harwood,[2,3] David Ackerley,[2,3] Michelle Kelly,[4] S. Vailala Matoto,[5] Patricia Hunt,[1] A. Jonathan Singh,[2,6]* Robert A. Keyzers.[1,2,*]

1 School of Chemical & Physical Sciences, and Centre for Biodiscovery, Victoria University of Wellington, PO Box 600, Wellington 6140, New Zealand; jenniramirezg@gmail.com, patricia.hunt@vuw.ac.nz

2 Maurice Wilkins Centre for Molecular Biodiscovery, University of Auckland, Private Bag 92019 Auckland, New Zealand

3 School of Biological Sciences, and Centre for Biodiscovery, Victoria University of Wellington, PO Box 600, Wellington 6140, New Zealand; hannahleeharwood@gmail.com, david.ackerley@vuw.ac.nz

4 Oceans Centre, National Institute of Water & Atmospheric Research, PO Box 9940, Auckland, New Zealand; michelle.kelly@niwa.co.nz

5 Ministry of Fisheries, Sopu, Vuna Road, Tongatapu, Kingdom of Tonga; vailalam@yahoo.com

6 Ferrier Research Institute, and Centre for Biodiscovery, Victoria University of Wellington, PO Box 600, Wellington 6140, New Zealand.

* Correspondence: jonathan.singh@vuw.ac.nz (AJS); robert.keyzers@vuw.ac.nz; Tel.: (+64 4 463 5117, RAK)

List of Figures

Figure S1 – HPLC chromatogram of Tongan Pseudoceratina cf. verrucosa purification (210 nm). S2
Figure S2 – ^{1}H NMR spectrum (CD_3OD, 600 MHz) of compound **1** S3
Figure S3 – ^{13}C NMR spectrum (CD_3OD, 151 MHz) of compound **1** S4
Figure S4 – HSQC spectrum (CD_3OD, 600 MHz) of compound **1** S5
Figure S5 – COSY spectrum (CD_3OD, 600 MHz) of compound **1** S6
Figure S6 – HMBC spectrum (CD_3OD, 600 MHz) of compound **1** S7
Figure S7 – (+)-HRESIMS of compound **1** S8
Figure S8 – (+)-HRESIMS/MS spectra of **1** using CID of 10 (a), 20 (b), 30 (c) and 40 (d) eV........ S9
Figure S9 – UV spectra extracted from HPLC chromatogram of compound **1** S10
Figure S10 – ^{1}H NMR spectrum (CD_3OD, 600 MHz) of hexadellin A S11
Figure S11 – ^{13}C NMR spectrum (CD_3OD, 151 MHz) of hexadellin A S12
Figure S12 – ^{1}H NMR spectrum (CD_3OD, 600 MHz) of purealidin B........ S13
Figure S13 – ^{13}C NMR spectrum (CD_3OD, 151 MHz) of purealidin B S14
Figure S14 – ^{1}H NMR spectrum (CD_3OD, 600 MHz) of purpuramine M S15
Figure S15 – ^{13}C NMR spectrum (CD_3OD, 151 MHz) of purpuramine M S16

List of Tables

Table S1 – NMR spectroscopic data of purpuramine R (**1**) (600/151 MHz, CD_3OD)........ S2

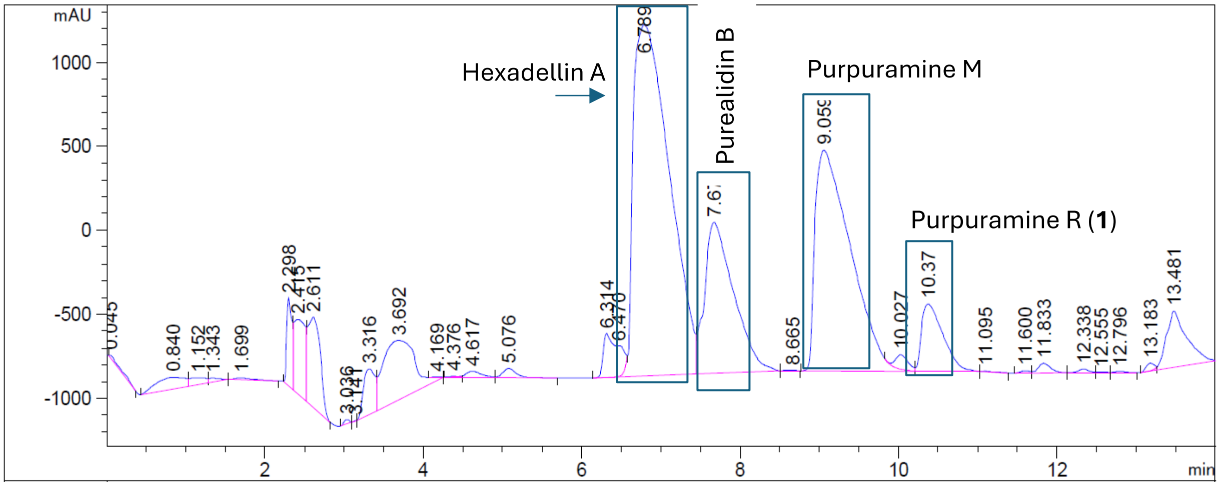

Figure S1 – HPLC chromatogram (210 nm) of Tongan *Pseudoceratina* cf. *verrucosa*, 30% $Me_2CO_{(aq)}$ fraction.

Table S1 – NMR spectroscopic data of purpuramine R (**1**) (600/151 MHz, CD_3OD)

**Position**	**¹³C**			**¹H**			**COSY**	**HMBC (¹H to ¹³C)**
	***δ* (ppm)**	**type**	**$^1J_{CH}$ (Hz)**	***δ* (ppm)**	**mult**	**J_H (Hz)**		
1	123.0	C		-				
2	151.7[a]	C		-				
3	108.8[a]	C		-				
4	154.7[a]	C		-				
5	106.8[a]	C		-				
6	134.5	CH	162	7.42	s			3, 4, 5, 7
7	25.6	CH_2	137	3.81	s, 2H			1, 2, 6, 8, 9
8	154.9[a]	C		-				
9	166.8[a]	C		-				
10	37.9	CH_2	134	3.58	t, 2H	6.7	11	9, 11, 12
11	30.5	CH_2	126	2.10	quin, 2H	6.4	10, 12	10, 12
12	72.2	CH_2	148	4.02	t, 2H	5.9	11	10, 11, 13
13	153.5[a]	C		-				
14/18	119.5	C		-				
15/17	134.6	CH	170	7.58	s, 2H			13, 14, 18, 19
16	136.0[a]	C		-				
19	28.8	CH_2	128	3.09	m, 2H		20	15/17, 16
20	67.7	CH_2	148	3.54	m, 2H		19	19, 21
21	53.6–53.7	$N^+(CH_3)_3$	148	3.20	s, 9H			20, 21
22	60.8	CH_3	145	3.80	s, 3H			4

[a]Detected by HMBC.

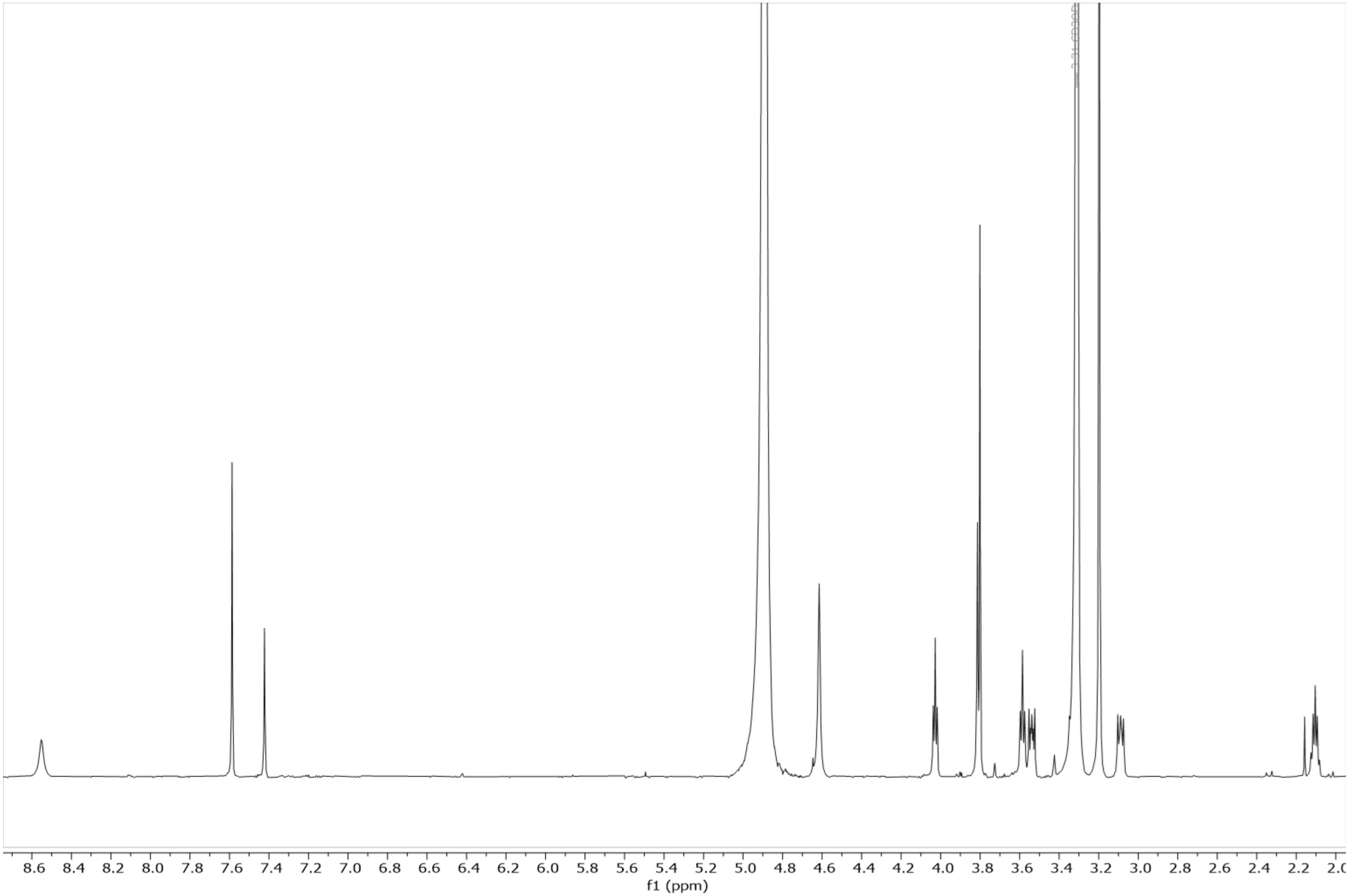

Figure S2 – ^{1}H NMR spectrum (CD_3OD, 600 MHz) of compound **1**

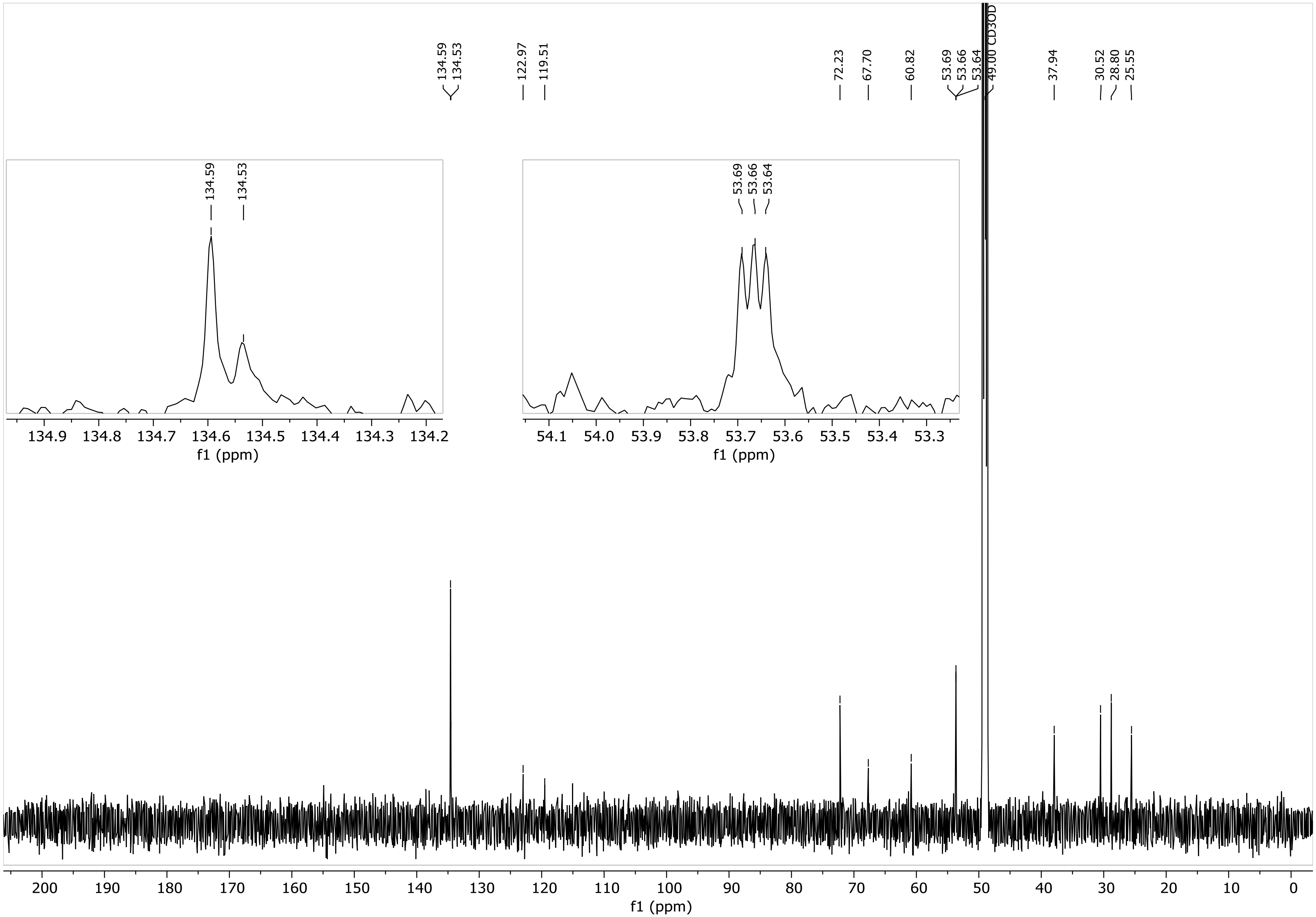

Figure S3 – ^{13}C NMR spectrum (CD_3OD, 151 MHz) of compound **1**

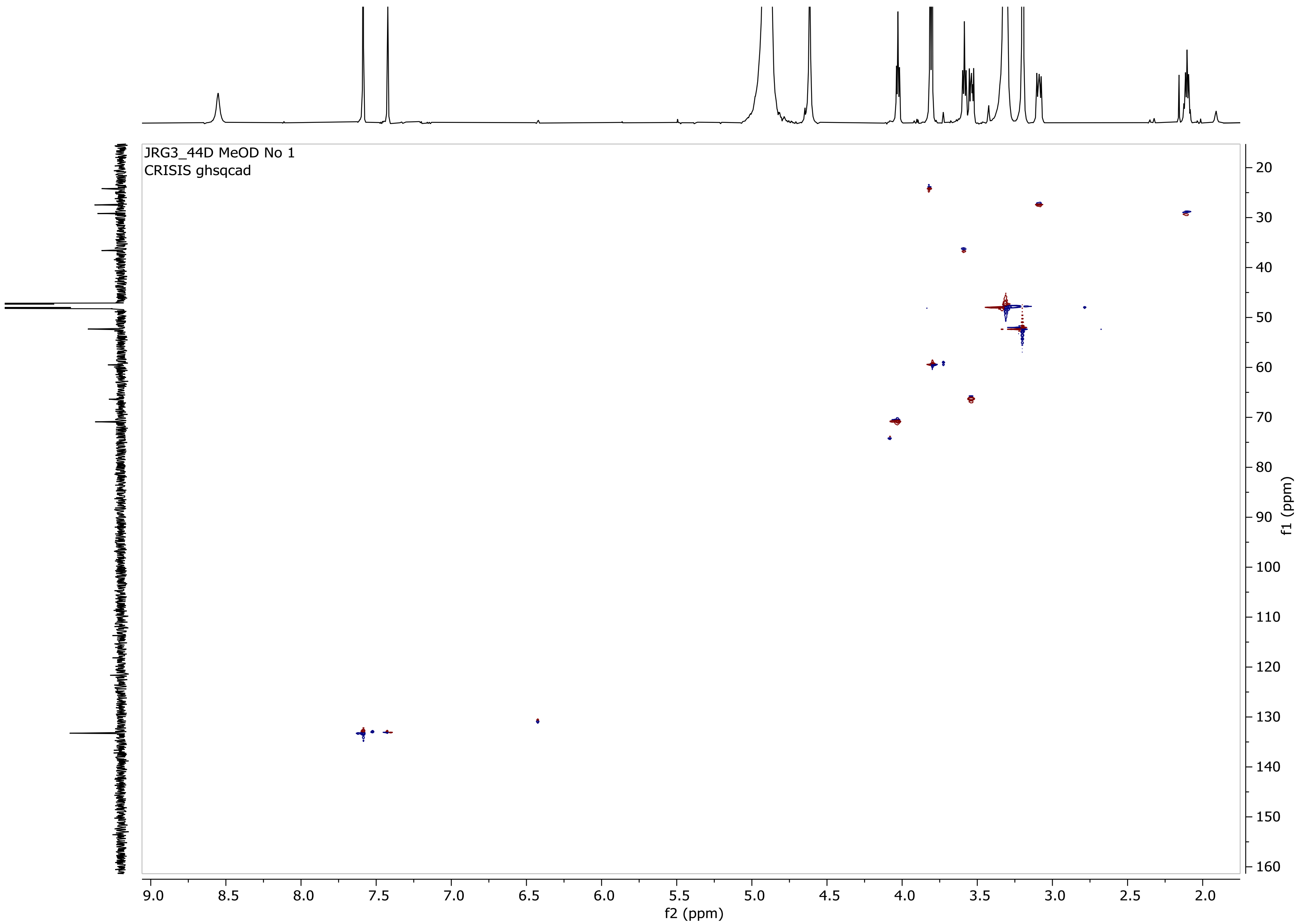

Figure S4 – HSQC spectrum (CD_3OD, 600 MHz) of compound **1**

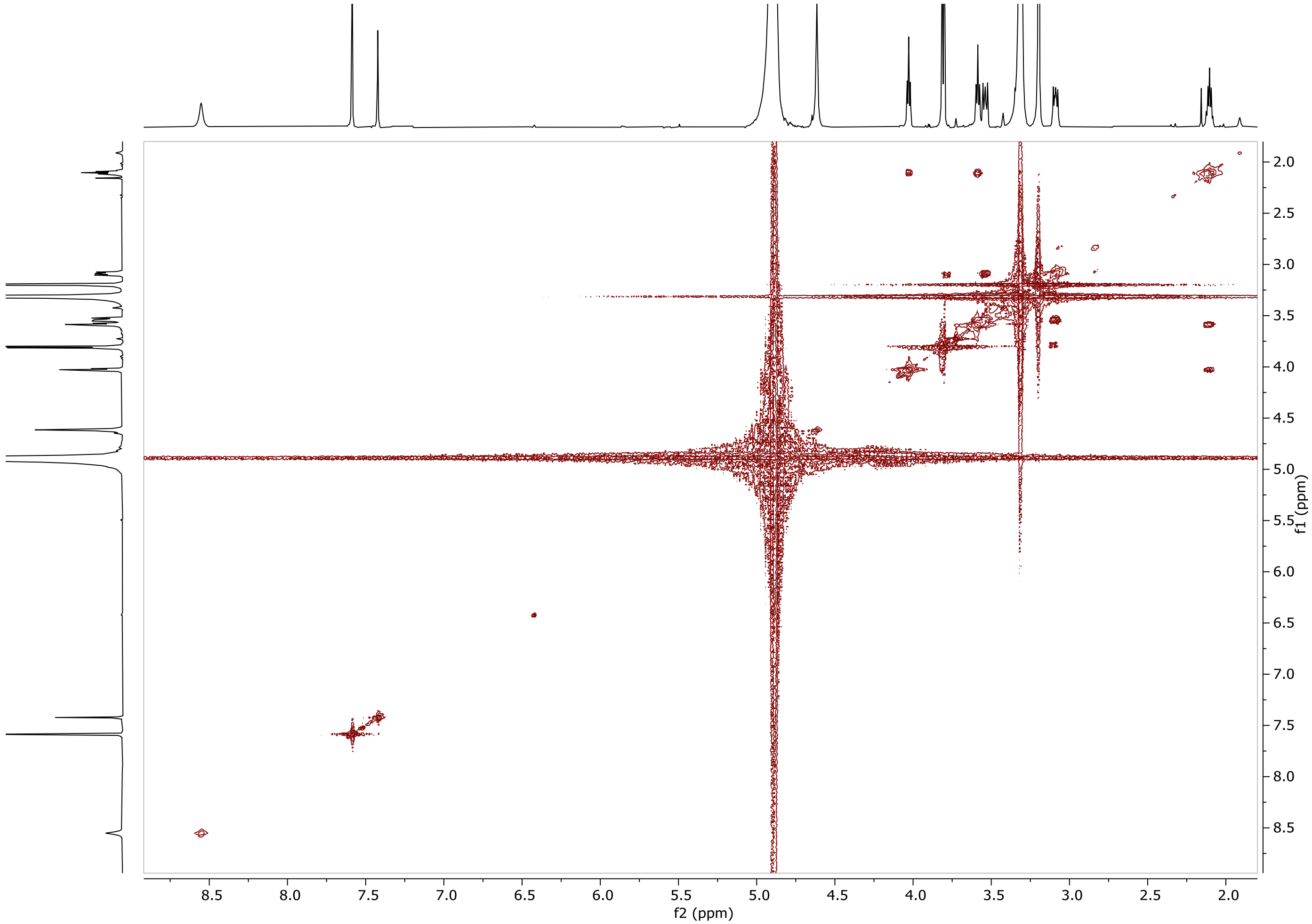

Figure S5 – COSY spectrum (CD_3OD, 600 MHz) of compound **1**

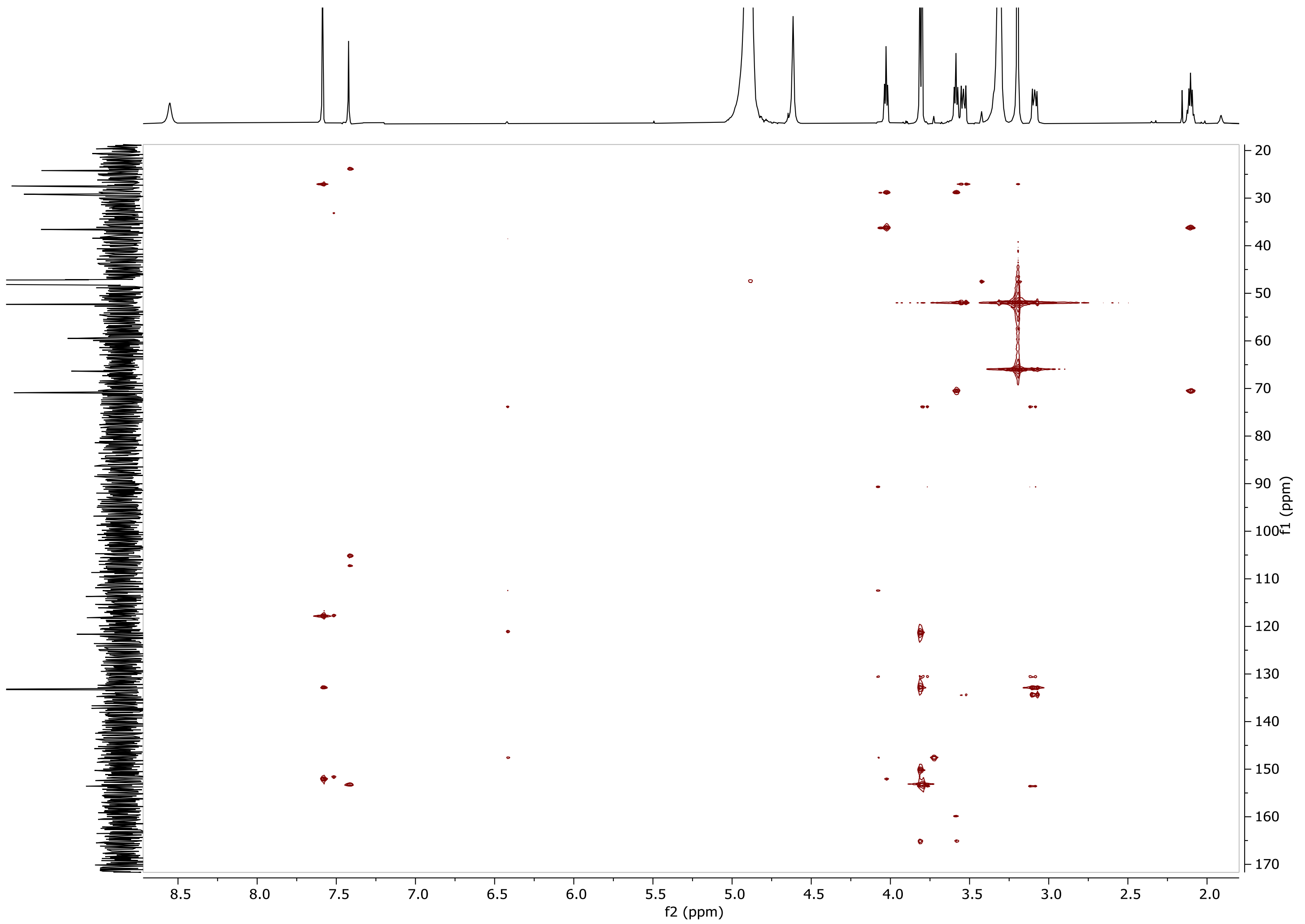

Figure S6 – HMBC spectrum (CD_3OD, 600 MHz) of compound **1**

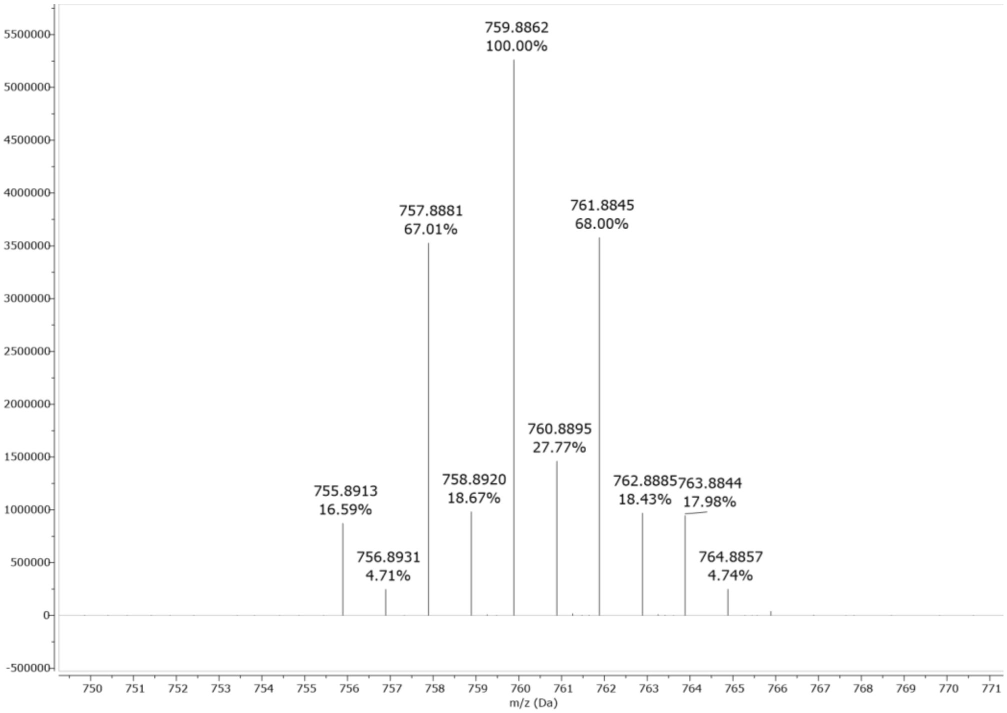

Figure S7 – (+)-HRESIMS of compound **1**

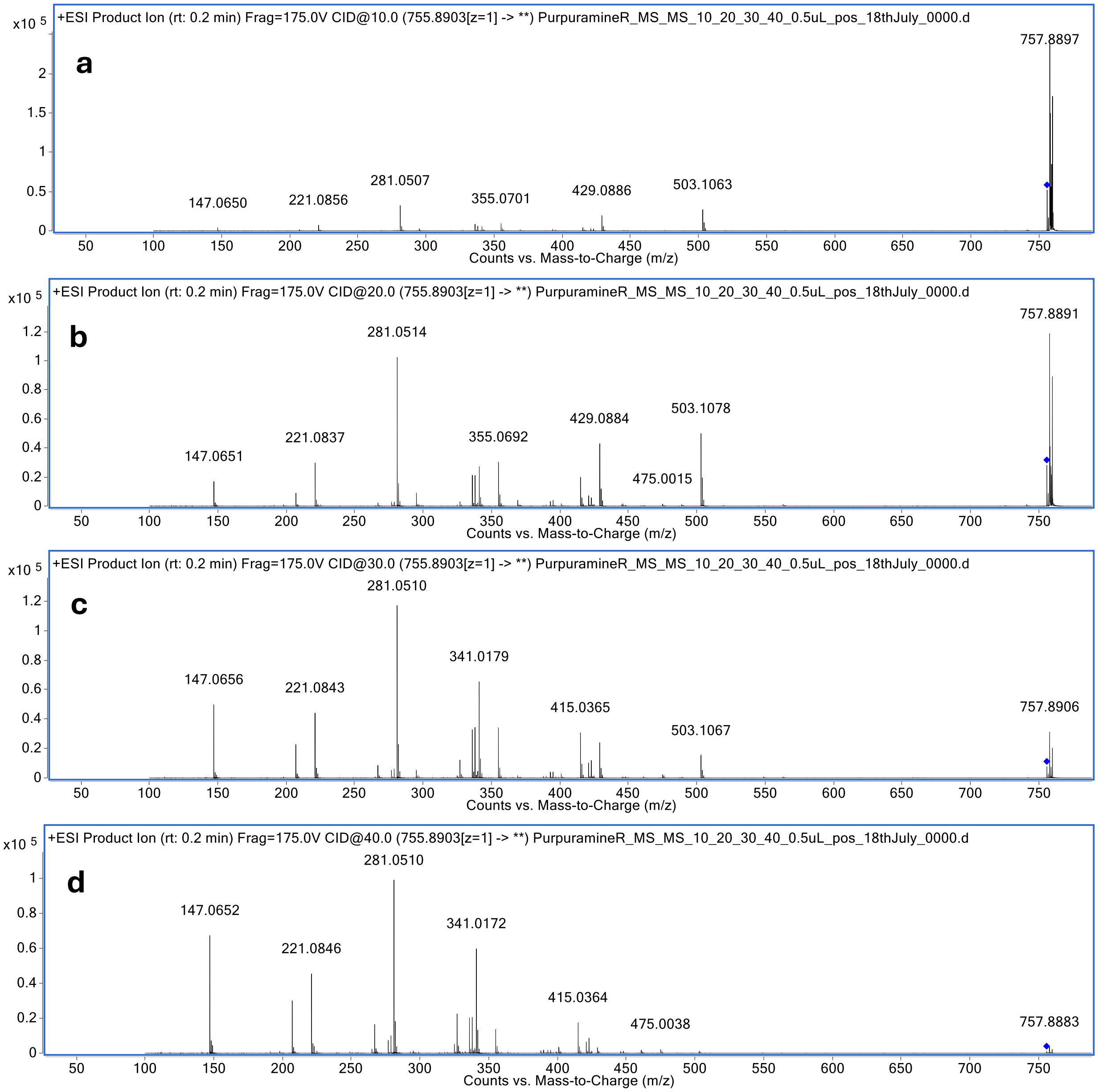

Figure S8 – (+)-HRESIMS/MS spectra of **1** using CID of 10 (a), 20 (b), 30 (c) and 40 (d) eV.

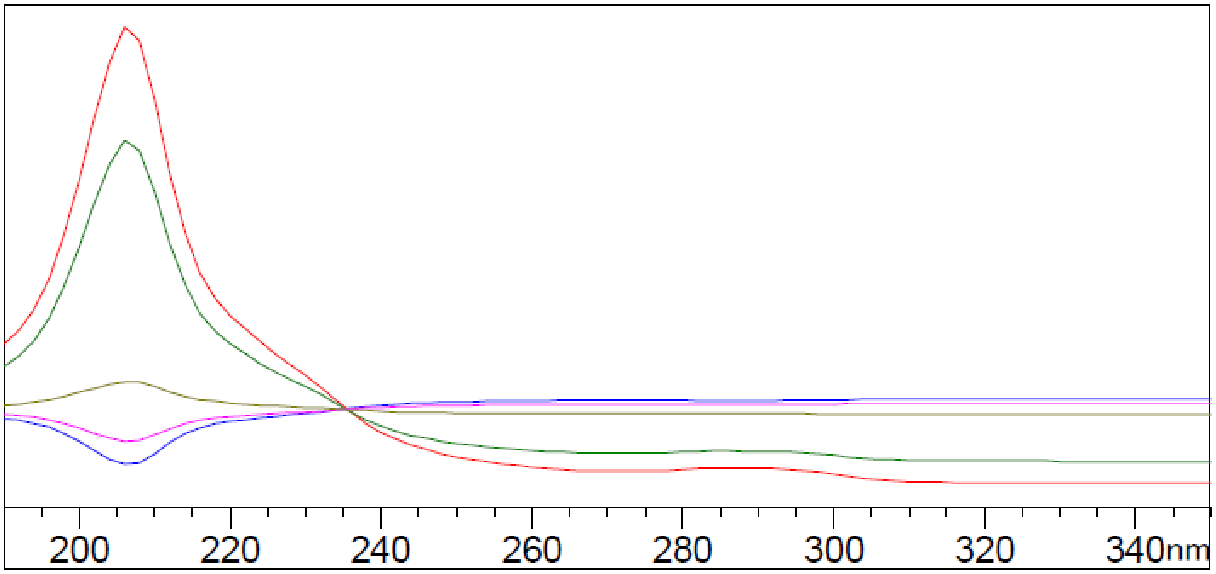

Figure S9 – UV spectra extracted from HPLC chromatogram of compound **1**

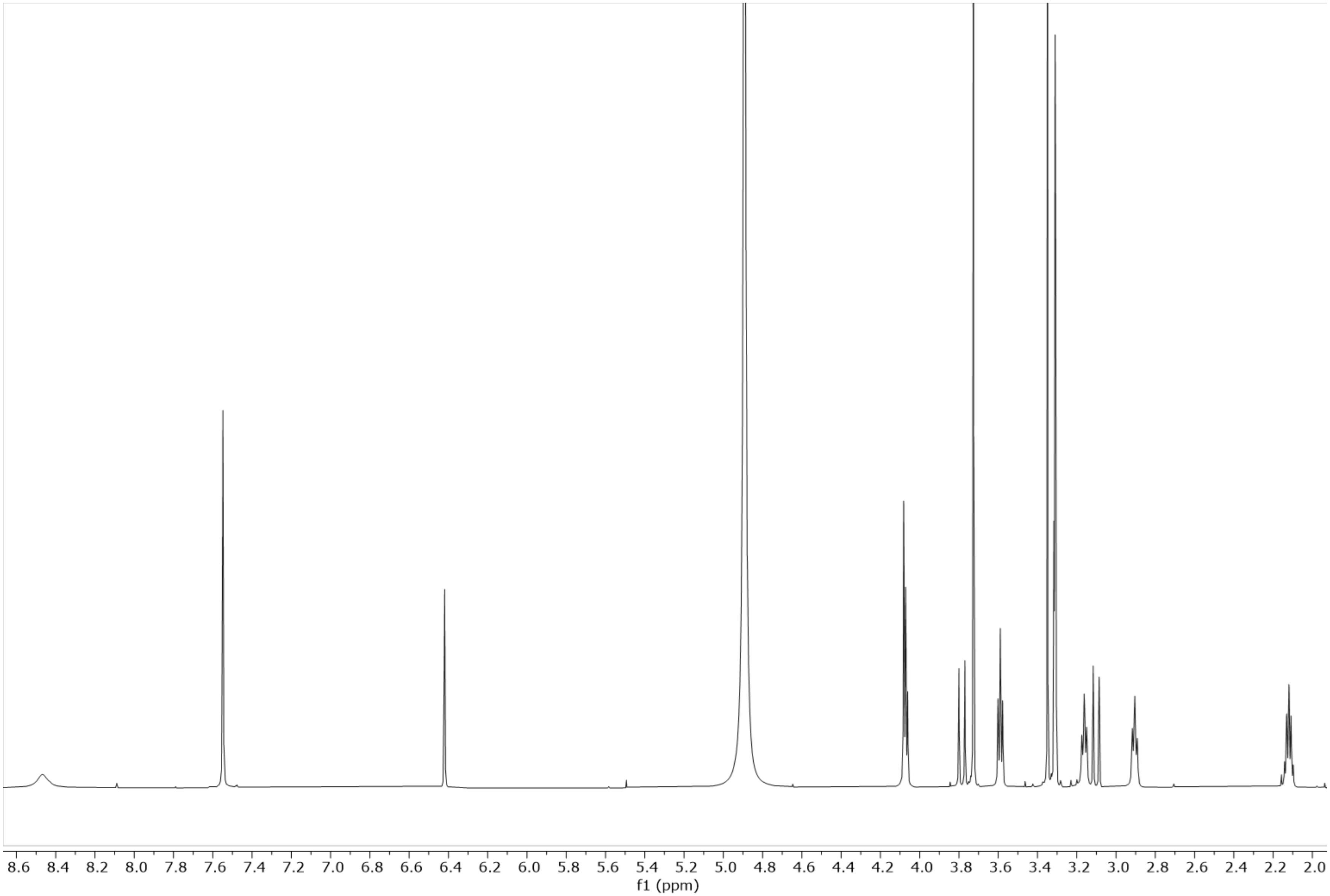

Figure S10 – ^{1}H NMR spectrum (CD_3OD, 600 MHz) of hexadellin A

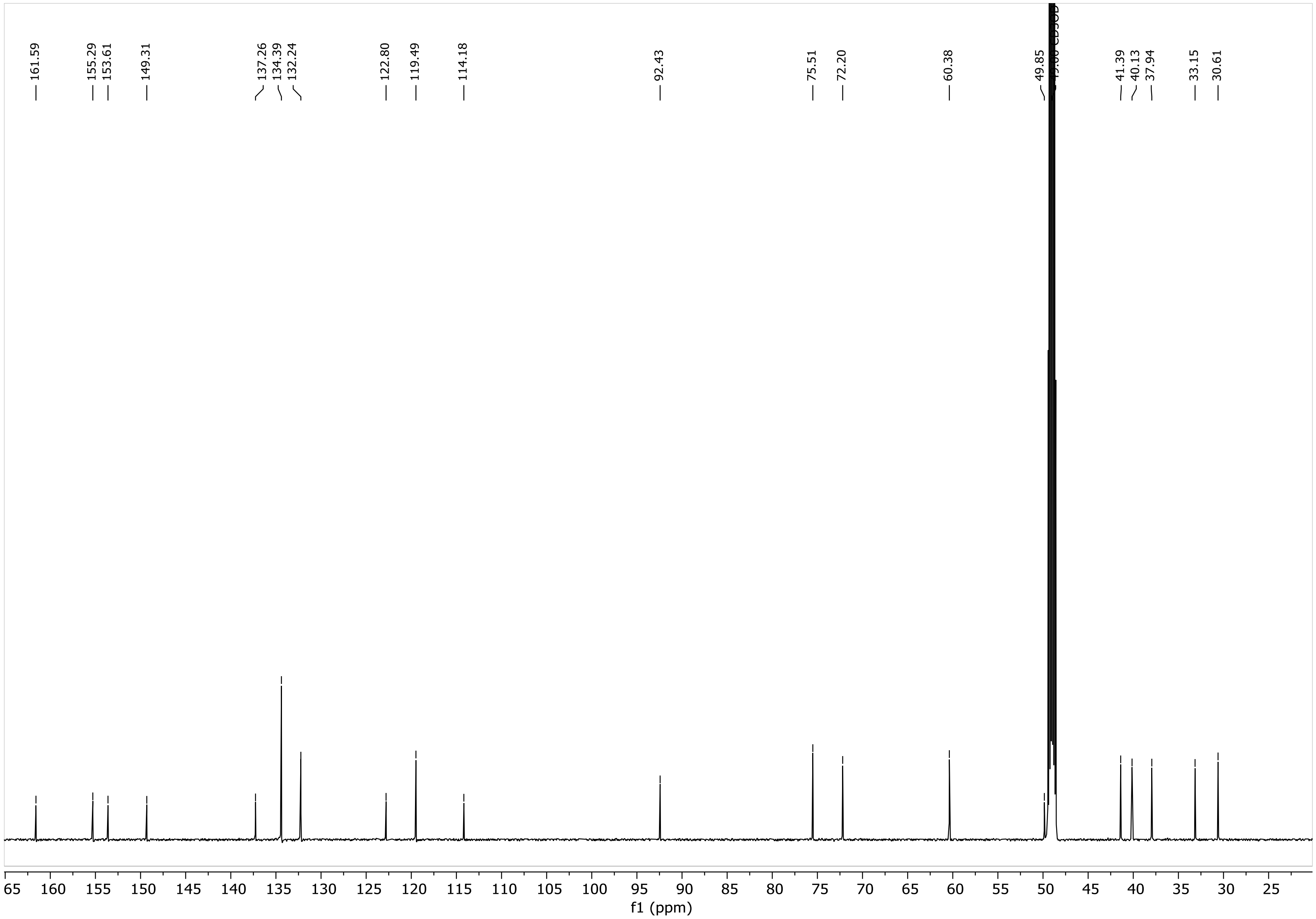

Figure S11 – ^{13}C NMR spectrum (CD_3OD, 151 MHz) of hexadellin A

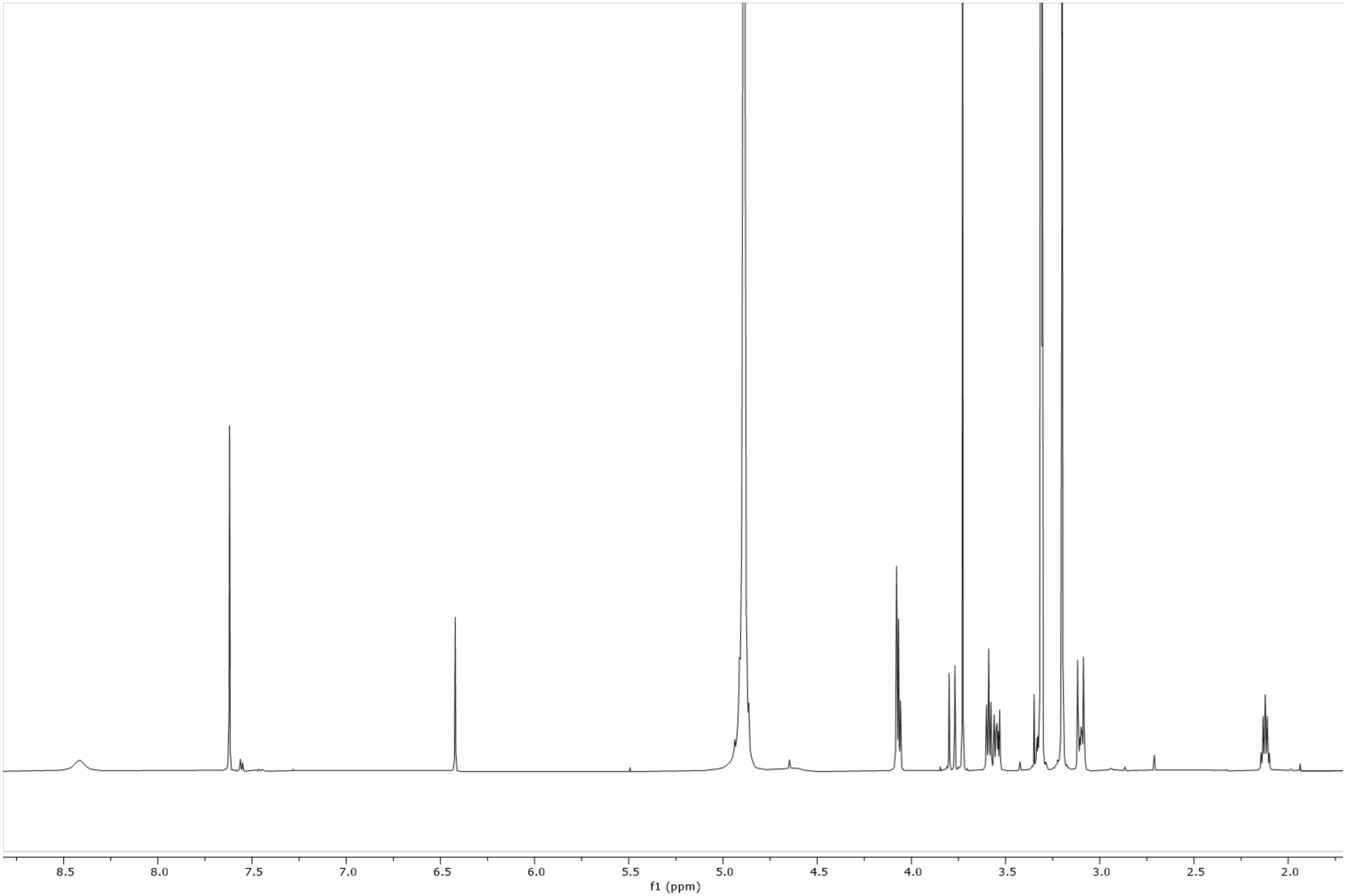

Figure S12 – ^{1}H NMR spectrum (CD_3OD, 600 MHz) of purealidin B

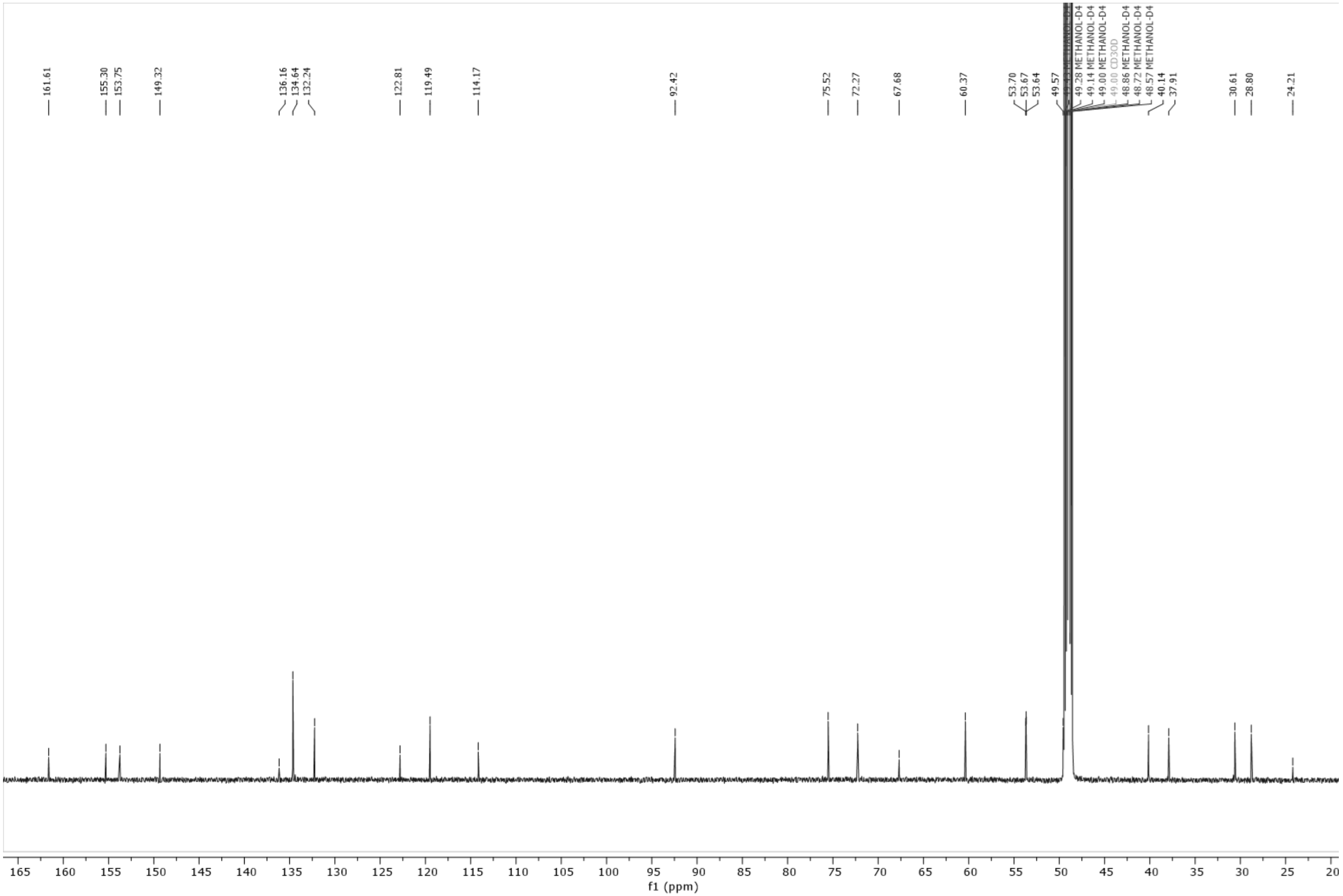

Figure S13 – ^{13}C NMR spectrum (CD_3OD, 151 MHz) of purealidin B

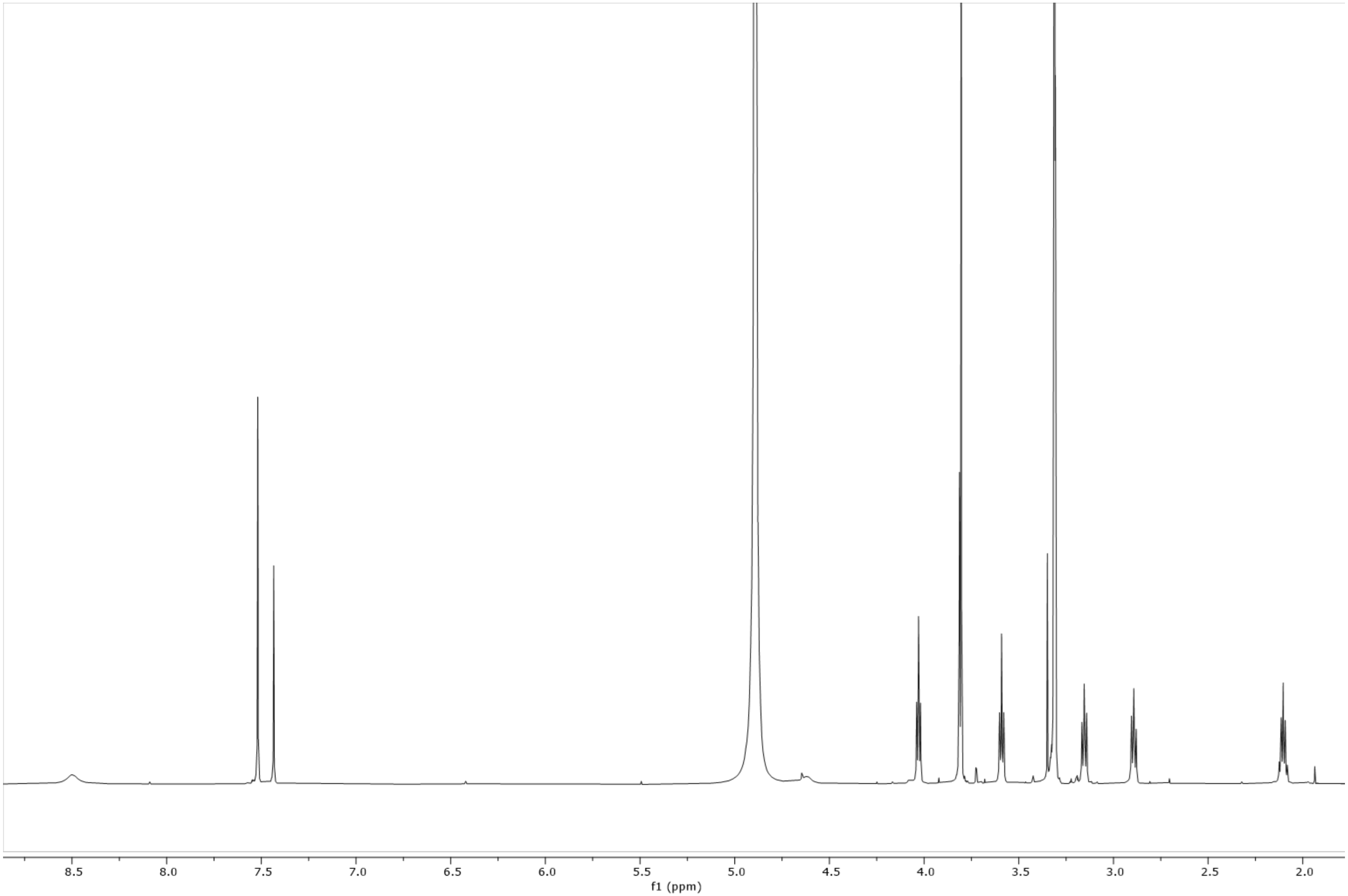

Figure S14 – ^{1}H NMR spectrum (CD_3OD, 600 MHz) of purpuramine M

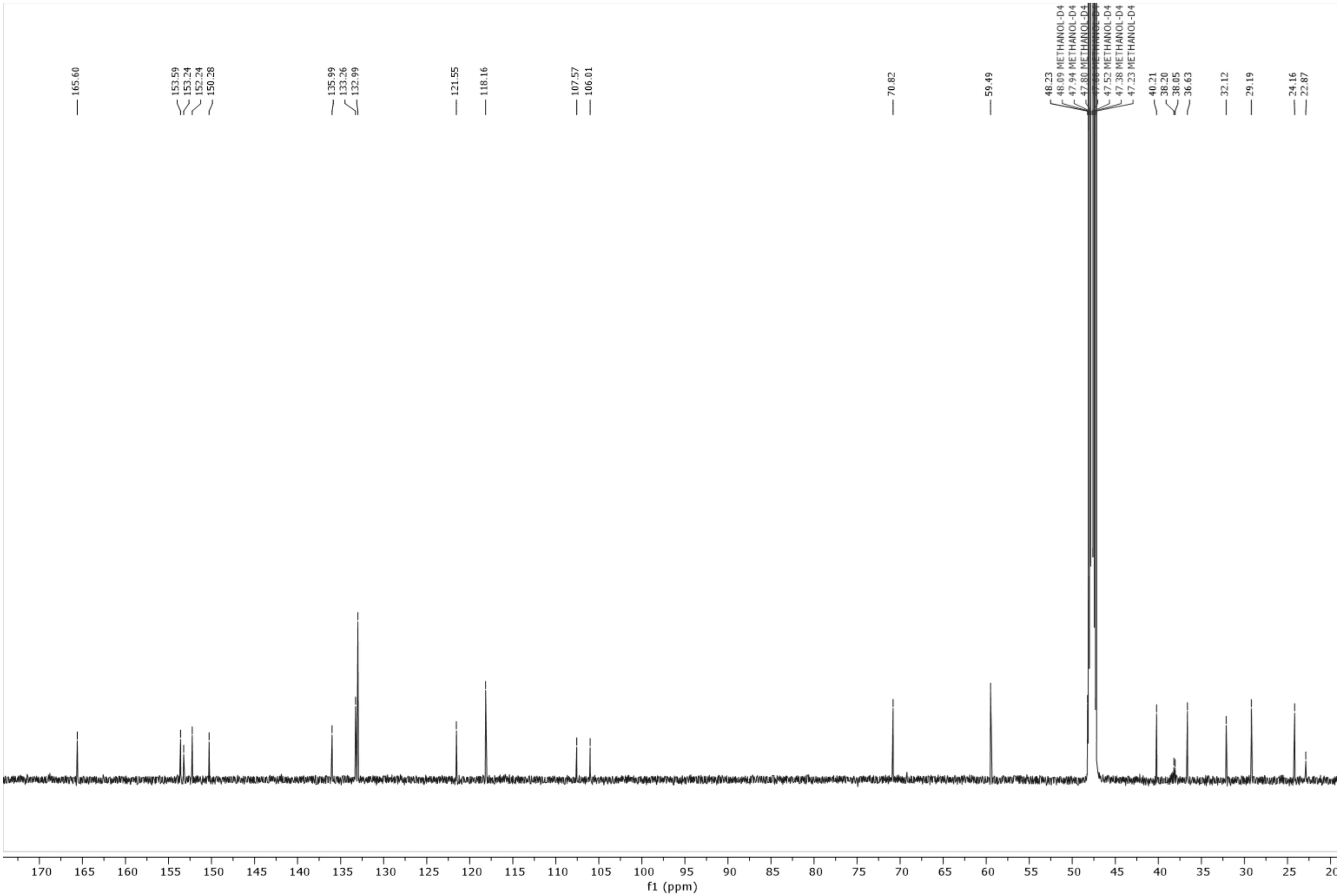

Figure S15 – ^{13}C NMR spectrum (CD_3OD, 151 MHz) of purpuramine M
